# Ingroup-Outgroup Bias in Contagious Yawning by Chimpanzees Supports Link to Empathy

**DOI:** 10.1371/journal.pone.0018283

**Published:** 2011-04-06

**Authors:** Matthew W. Campbell, Frans B. M. de Waal

**Affiliations:** Living Links Center, Yerkes National Primate Research Center, Emory University, Atlanta, Georgia, United States of America; Université Pierre et Marie Curie, France

## Abstract

Humans favor others seen as similar to themselves (ingroup) over people seen as different (outgroup), even without explicitly stated bias. Ingroup-outgroup bias extends to involuntary responses, such as empathy for pain. However, empathy biases have not been tested in our close primate relatives. Contagious yawning has been theoretically and empirically linked to empathy. If empathy underlies contagious yawning, we predict that subjects should show an ingroup-outgroup bias by yawning more in response to watching ingroup members yawn than outgroup. Twenty-three chimpanzees (*Pan troglodytes*) from two separate groups watched videos of familiar and unfamiliar individuals yawning or at rest (control). The chimpanzees yawned more when watching the familiar yawns than the familiar control or the unfamiliar yawns, demonstrating an ingroup-outgroup bias in contagious yawning. These results provide further empirical support that contagious yawning is a measure of empathy, which may be useful for evolutionary biology and mental health.

## Introduction

Humans (*Homo sapiens*) favor other humans seen as belonging to their own group (ingroup) over humans seen as belonging to different social groups (outgroup), even in absence of explicitly stated bias [1], [2]. Recently, these biases have been extended to differential brain activity during empathy for pain. Specific brain areas, most notably the anterior cingulate cortex (ACC) and anterior insula, activated during functional magnetic resonance imaging (fMRI) both when subjects experienced pain and when another person present experienced pain, whereas other areas activated only during the direct sensation of pain [3]. Singer et al. [3] interpreted these findings as humans sharing the affective or emotional aspect of pain with others, but not the physical sensation of pain. Extending these findings to bias, two studies presented visuals of painful experiences to human ingroup and outgroup members (as defined by race) while using fMRI to examine brain activity [4], [5]. Xu et al. [4] found greater activity in the ACC in response to ingroup empathy for pain than outgroup, and Mathur et al. [5] found differences in the medial pre-frontal cortex, indicating a role of cognitive appraisal. During transcranial magnetic stimulation (TMS) human subjects watched videos of needles penetrating the hand of ingroup or outgroup members, also defined by race [6]. The subjects showed a greater empathic response (in the form of resonant corticospinal inhibition) to the ingroup than outgroup stimuli. Most interesting, subjects also saw needles penetrating a hand that had been artificially colored violet, removing race cues. The subjects responded with a greater empathic response toward the violet hand than the outgroup hand, yielding a pattern of ingroup > violet > outgroup.

All three studies showed that humans have differential empathic responses to pain based upon group status, indicating ingroup-outgroup bias. We wanted to explore whether ingroup-outgroup bias is present in contagious yawning. Lehmann [7] and Preston and de Waal [8] both hypothesized that empathy is the mechanism underlying contagious yawning. The idea is that yawns are contagious for the same reason that smiles, frowns, and other facial expressions are contagious. The mechanism that allows someone to reflexively mimic a smile [9] is thought to also allow for reflexive mimicry of yawns. In this article, we use the definition of empathy supplied by Preston & de Waal [8], in which empathy is a term for a broad category of resonant emotional responses comprising a continuum from basic forms, such as emotional contagion, to complex forms, such as cognitive empathy.

The link between empathy and contagious yawning has empirical support. Humans who performed better at self-recognition and theory-of-mind, two abilities that contribute to complex empathy, performed more contagious yawning [10]. In gelada baboons (*Theropithecus gelada*), the closer the social bond between individuals, the more likely they would yawn when the other yawned [11]. This finding is consistent with the observation that empathy is more pronounced the closer the relationship between individuals [8], [12]. Also informative are the negative relationships. Two conditions, schizotypy [10] and the autism quotient [13], [14], are associated with decreased contagious yawning, possibly to the point of being absent in autism. Both of these conditions are associated with atypical empathy functioning.

Contagious yawning has been documented in five mammalian species: humans [10], [13], [14], [15], [16], [17], chimpanzees [18], [19], stumptail macaques (*Macaca arctoides*) [20], gelada baboons [11], and domestic dogs (*Canis familiaris*) [21], [22], although some of the interpretations differ. Because of its relevance to human mental health, evolutionary biology, and as a potential low-cost complement to other measures, contagious yawning is a useful and perhaps under-utilized tool for studying empathy functioning.

Our hypothesis was that if empathy is the mechanism underlying contagious yawning, then contagious yawning should show the same biases as other measures of empathy, specifically the ingroup-outgroup bias. We tested two groups of captive chimpanzees by showing them yawn and control videos of their own group and the strange group. Chimpanzees form communities that are territorial and exclude neighboring individuals and communities [23]. Thus, for chimpanzees, strangers are outgroup by default. Evidence for an ingroup-outgroup bias would be if chimpanzees yawned more in response to watching familiar individuals yawning than strangers. Studying chimpanzees also allows us to test whether human ingroup-outgroup empathy bias is rooted in evolved mechanisms assessing social closeness, familiarity, and group status.

## Methods

Ethics: The experiment presented was approved by the Institutional Animal Care and Use Committee of Emory University (#083-2008Y) and was conducted in accordance with the “Guidelines for the treatment of animals in behavioural research and teaching” by the Animal Behavior Society/Association for the Study of Animal Behaviour and the Weatherall report on “The use of non-human primates in research”. We used voluntary testing to minimize stress on the subjects.

The subjects were 23 adult chimpanzees (*Pan troglodytes*, age 10–46, 4 m/19 f) housed in two groups of 12 at the Yerkes National Primate Research Center (one individual was not tested due to a lack of attention). The 4 males and 19 females ranged in age from 10 to 46. The chimpanzees lived in large outdoor enclosures (group 1: 711 m^2^; group 2: 528 m^2^) with indoor sleeping quarters and could not see each other. Group 1 had an additional indoor testing building. Chimpanzees were tested indoors or outdoors depending upon where they approached the video player. Chimpanzees saw only one stimulus per day between 10.00 h and 13.30 h.

We recorded spontaneous yawns from both groups of chimpanzees with a PV-GS500 (Panasonic, Inc.) digital video camera. We obtained yawns from seven individuals from each group, selected one yawn from each individual based on the quality of the segment (e.g., viewing angle, only one individual in the frame, etc.), and edited each yawn clip to 9 s using iMovie HD (Apple, Inc.). From the same footage we selected 9 s control segments from each of the same individuals at rest. By using the same footage we were able to select control clips with virtually identical viewing angles, postures, background compositions, lighting, etc. We included 1 s of green screen between each clip and assembled them into a yawn video and a control. Each clip was shown once before repeating, and the order within a set was randomized on the condition that the same clip could not be shown consecutively.

The videos were presented on an iPod Touch (Apple, Inc.) with a 7.5×5 cm screen. The iPod was presented on its own to a chimpanzee when other individuals were not within view. When more than one individual was within view, we placed the iPod up against an opaque container with an eyehole at the opposite end. The hole was small enough that only one chimpanzee could see through it at a time. Chimpanzees were tested alone, in small groups, or with the entire group based upon their comfort. Some individuals were at ease being alone and were tested closed in a room indoors. Most individuals were more comfortable when in a group. Small groups and individual testing was also needed to ensure that low-ranking individuals could get access to the video player. Regardless of whether an individual was alone or in a group, the small screen of the iPod and the container with the eyehole ensured that we could present the video to one subject at a time.

Each chimpanzee was exposed to the videos for a total of 20 minutes on one or more days, depending upon the interest and cooperativeness of each individual. Some chimpanzees reached 20 min in one session, but most needed more than one day of testing. Timing for each session started after the chimpanzee first looked at the video. The chimpanzee was then free to watch the video or not as it chose, however the entire 20 min of recording was after observing the video and thus reflects the influence of viewing the video on the chimpanzee. Within each condition, ingroup or outgroup, the order of yawn and control videos was counter balanced (as close as possible due to the odd number of subjects). However, all subjects saw the ingroup videos before switching to the outgroup videos, as the outgroup exposure was a follow-up. Whereas we can envision order effects when the videos were of the same subjects (e.g., there could be less attention on the second or subsequent viewings), we cannot envision *a priori* reasons for order effects to occur when the stimuli changed to something novel.

A digital video camera recorded each session. We coded each session for the number of yawns by each chimpanzee after the first attention toward the video. We also coded the amount of attention each individual paid to the video in s. We analyzed the results using PASW Statistics 18.0 for Macintosh (SPSS, IBM Inc.) according to our previous recommendations [24]. Only planned comparisons of theoretical importance were conducted (paired *t*-tests unless indicated). Values reported are means ± SEM, and all statistics were two-tailed.

## Results

**Figure 1:**
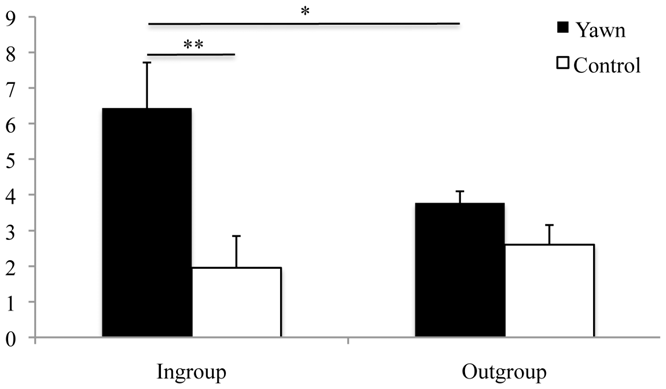
Mean rate of yawning + SEM in response to the four different videos. ** *p* = 0.002, * *p* = 0.012.

The chimpanzees yawned more frequently in response to the ingroup yawn video than to the ingroup control (*t*_22_ = 3.61, *p* = 0.002, *d* = 1.05, Figure 1). There was no difference in rate of yawning between the outgroup yawn and control videos (*t*_22_ = 1.40, *p* = 0.175, *d* = 0.34, Figure 1), but the chimpanzees yawned more in response to the ingroup yawn video than the outgroup yawn video (*t*_22_ = 2.73, *p* = 0.012, *d* = 0.51, Figure 1).

There were no differences in yawning between females (ingroup: 6.53±1.40, outgroup: 3.68±1.01) and males (ingroup: 6.00±3.24, outgroup: 4.25±1.80) for either stimulus (both: independent samples *t*_21_<1.0, NS). Fourteen individuals saw clips of themselves yawning in the ingroup video (1 in 7 clips), but these individuals yawned at similar rates (5.21±1.42) as the 9 individuals who did not (8.33±2.28; independent samples *t*_21_ = 1.23, *p* = 0.23, *d*  = 0.54). There were no relationships between the amount of yawning and the number of sessions (1–5) needed to reach 20 min of exposure (ingroup: Pearson's *r* = −0.13, NS; outgroup: Pearson's *r* = 0.13, NS).

**Figure 2:**
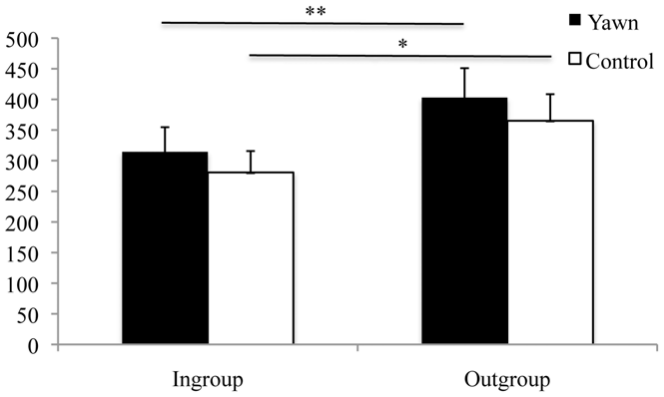
Mean amount of attention + SEM in s paid to the different videos. ** *p* = 0.008, * *p* = 0.018.

The subjects paid similar amounts of attention to the yawn and control videos for both conditions (ingroup: *t*_22_<1.0, NS; outgroup *t*_22_ = 1.13, *p* = 0.27, *d* = 0.18, Figure 2). However, the subjects paid more attention to the outgroup than the ingroup videos (yawn: *t*_22_ = 2.94, *p* = 0.008, *d* = 0.53; control: *t*_22_ = 2.56, *p* = 0.018, *d* = 0.48, Figure 2). There were no correlations between attention toward the yawn video and the number of yawns for either condition (ingroup: Pearson's *r* = 0.26, *p* = 0.23; outgroup: Pearson's *r* = 0.13, NS).

## Discussion

The chimpanzees yawned more in response to the familiar yawn video than the familiar control, demonstrating contagious yawning. However, the video of unfamiliar chimpanzees had no detectable effect, as the difference in yawning between the yawn and control videos was nonsignificant. Critically, the chimpanzees yawned more in response to the familiar yawns than the unfamiliar yawns, demonstrating ingroup-outgroup bias. This bias supports the hypothesis that empathy is the mechanism underlying contagious yawning.

The link between empathy and contagious yawning is further supported by our data on attention. The chimpanzees actually watched the videos of unfamiliar individuals more than the videos of familiar individuals. They attended more to the unfamiliar yawns, but yawned more to the familiar yawns. This finding rules out attention *per se* as a mediating factor and supports the idea that social identification with the stimuli influenced the rate of contagion.

Even though all of the ingroup videos were presented before the outgroup videos, we can think of no *a priori* reasons for an order effect. The attention data show that the chimpanzees did not lose interest in the videos since they watched the outgroup videos more than the ingroup videos. There is no evidence nor are there suggestions in the literature that contagious yawning is transient and fluctuates over time. These same subjects were previously tested and showed contagious yawning [19], so contagion seems to be an enduring behavior. The rate of yawning toward all of the control videos has remained the same over three years (2007–2010) and three different stimuli, suggesting no change in baseline rates of yawning. The more pertinent order effect would be between the yawn and control videos within a stimulus type (i.e., ingroup or outgroup), but these were always counter-balanced.

In contrast to chimpanzees, humans [10], [13], [14], [15], [16], [17] and dogs [21], [22] have shown contagion in response to watching unfamiliar individuals yawn. Some different variables may explain this. First, we cannot rule out that our sample size, large by chimpanzee standards, was too small to detect a significant difference. Chimpanzees may indeed yawn contagiously in response to unfamiliar individuals, but if so the magnitude of the effect is probably small and would require more subjects to detect statistically. A similar situation occurred in the first study of yawn contagion in chimpanzees [18], which had too small of a sample size to detect contagious yawning at the population level (the significant effects were at the individual level). In addition, Anderson et al. [18] did test for ingroup-outgroup bias, but since they could not detect a population-level effect for contagious yawning overall, they did not detect a difference between these stimuli. Larger samples of chimpanzees have shown population-level contagious yawning and an ingroup-outgroup effect ([19] and the present study). It may take an even larger sample than the one we had available to detect yawn contagion in response to unfamiliar chimpanzees.

We should also be mindful of social structure, as we may have two different factors at work: familiarity and group membership. Chimpanzees are territorial and aggressive toward neighboring communities [23]. Since all members of a community know each other, for chimpanzees, unfamiliar individuals are by definition outgroup individuals. Humans, at some point in our evolution, gained the ability to include unfamiliar individuals in our ingroup. Therefore, humans do not necessarily view strangers as belonging to an outgroup. Pet dogs are accustomed to interacting with unfamiliar humans, and sometimes unfamiliar dogs, in positive ways. Possibly, we artificially selected dogs to, like us, have disassociated familiarity and group status, but this needs testing.

Exposed to artificial stimuli that transcend the ingroup-outgroup distinction, chimpanzee yawn contagion shows patterns similar to those of brain imaging studies of empathy. Chimpanzees yawned in response to 3D computer-animated chimpanzees yawning [19]. These animations were not familiar individuals, yet they stimulated contagious yawning. Chimpanzees seem to process animations the same way they process pictures of chimpanzees [25], but the inherent artificiality of the animations may have prevented them from being processed as outgroup individuals. This finding is similar to the greater empathy of humans to pain inflicted on a hand artificially colored purple than a hand of an other-race individual present in society [6]. Thus, animations and artificial stimuli may allow us to distinguish between and test the variables of familiarity and group status, in humans and nonhumans alike.

Contagious yawning in humans has not yet been tested for biases, including social closeness [11] and ingroup-outgroup bias, but we would expect similar responses. Contagious yawning has several advantages as a measure of empathy given its low cost, high portability, and applicability to multiple species, which may make it a useful complement to physiological, questionnaire, and mental health diagnostic based measures of empathy. Given that chimpanzees exhibit both altruism [26] and extreme violence [23] toward others, studying how and when empathy is engaged may tell us about how humans switch between these two extremes as well.

## References

[1] Taijfel H (1970). Experiments in intergroup discrimination.. Scientific American.

[2] Amodio DM (2008). The social neuroscience of intergroup relations.. European Review of Social Psychology.

[3] Singer T, Seymour B, O'Doherty J, Kaube H, Dolan RJ (2004). Empathy for pain involves the affective but not sensory components of pain.. Science.

[4] Xu X, Zuo X, Wang X, Han S (2009). Do you feel my pain? Racial group membership modulates empathic neural responses.. Journal of Neuroscience.

[5] Mathur VA, Harada T, Lipke T, Chiao JY (2010). Neural basis of extraordinary empathy and altruistic motivation.. NeuroImage.

[6] Avenanti A, Sirigu A, Aglioti SM (2010). Racial bias reduces empathic sensorimotor resonance with other-race pain.. Current Biology.

[7] Lehmann HE (1979). Yawning: A homeostatic reflex and its psychological significance.. Bulletin of the Menninger Clinic.

[8] Preston SD, de Waal FBM (2002). Empathy: Its ultimate and proximate bases.. Behavioral and Brain Sciences.

[9] Dimberg U, Thunberg M, Elmehed K (2000). Unconscious facial reactions to emotional facial expressions.. Psychological Science.

[10] Platek SM, Critton SR, Myers TE, Gallup GG (2003). Contagious yawning: the role of self-awareness and mental state attribution.. Cognitive Brain Research.

[11] Palagi E, Leone A, Mancini G, Ferrari PF (2009). Contagious yawning in gelada baboons as a possible expression of empathy.. Proceedings of the National Academy of Sciences.

[12] Langford DJ, Crager SE, Shehzad Z, Smith SB, Sotocinal SG (2006). Social modulation of pain as evidence for empathy in mice.. Science.

[13] Senju A, Maeda M, Kikuchi Y, Hasegawa T, Tojo Y (2007). Absence of contagious yawning in children with autism spectrum disorder.. Biology Letters.

[14] Giganti F, Esposito Ziello M (2009). Contagious and spontaneous yawning in autistic and typically developing children.. Current Psychology Letters.

[15] Provine RR (1986). Yawning as a stereotyped action pattern and releasing stimulus.. Etholoy.

[16] Provine RR (1989). Faces as releasers of contagious yawning: An approach to face detection using normal humans subjects.. Bulletin of the Psychonomic Society.

[17] Anderson JR, Meno P (2003). Psychological influences on yawning in children.. Current Psychology Letters.

[18] Anderson JR, Myowa-Yamakoshi M, Matsuzawa T (2004). Contagious yawning in chimpanzees.. Proceedings of the Royal Society of London Biological Sciences.

[19] Campbell MW, Carter JD, Proctor D, Eisenberg ML, de Waal FBM (2009). Computer animations stimulate contagious yawning in chimpanzees.. Proceedings of the Royal Society of London Biological Sciences.

[20] Paukner A, Anderson JR (2006). Video-induced yawning in stumptail macaques (*Macaca arctoides*).. Biology Letters.

[21] Joly-Mascheroni RM, Senju A, Shepherd AJ (2008). Dogs catch human yawns.. Biology Letters.

[22] Harr A, Gilbert V, Phillips K (2009). Do dogs (*Canis familiaris*) show contagious yawning?. Animal Cognition.

[23] Nishida T, Hiraiwa-Hasegawa M, Hasegawa T, Takahata Y (1985). Group extinction and female transfer in wild chimpanzees in the Mahale National Park, Tanzania.. Zeitschrift für Tierpsychologie.

[24] Campbell MW, de Waal FBM, Walusinski O (2010). Methodolical problems in the study of contagious yawning.. The mystery of yawning in physiology and disease.

[25] Parr LA, Waller BM, Heintz M (2008). Facial expression categorization by chimpanzees using standardized stimuli.. Emotion.

[26] Warneken F, Hare B, Melis AP, Hanus D, Tomasello M (2007). Spontaneous altruism by chimpanzees and young children.. PLoS Biology.

